# The neurobiological basis of the awe experience in affective disorders: an exploratory EEG study

**DOI:** 10.3389/fnsys.2026.1805778

**Published:** 2026-06-04

**Authors:** Elena Bondi, Flavia Carbone, Giandomenico Schiena, Yara Massalha, MariaPia Marra, Lorena Di Consoli, Maddalena Mazzocut-Mis, Andrea Gaggioli, Alice Chirico, Paolo Brambilla, Eleonora Maggioni

**Affiliations:** 1 Department of Pathophysiology and Transplantation, Università degli Studi di Milano, Milan, Italy; 2 Department of Electronics, Information and Bioengineering, Politecnico di Milano, Milan, Italy; 3 Department of Neurosciences and Mental Health, Fondazione IRCCS Ca' Granda Ospedale Maggiore Policlinico, Milan, Italy; 4 Department of Cultural Heritage and Environment, Università degli Studi di Milano, Milan, Italy; 5 Research Center in Communication Psychology (PSICOM), Università Cattolica del Sacro Cuore, Milan, Italy

**Keywords:** affective disorders, awe, EEG, neural activity, VR

## Abstract

**Introduction:**

Affective disorders (ADs) are characterized by profound emotional processing deficits involving disrupted neural network activity and connectivity, particularly within the default mode network and fronto-temporal circuits, with abnormalities in theta and alpha oscillatory patterns. While current treatments primarily target mood symptoms, emotional processing impairments often persist and predict relapses. Awe, a complex self-transcendent emotion, may counteract such deficits through its capacity to reduce rumination and enhance positive affect. However, the neural correlates of awe experiences in clinical populations remain unexplored.

**Objective:**

For the first time, this exploratory study investigated the electroencephalographic (EEG) correlates of awe induced by validated virtual reality (VR) scenarios in individuals with ADs compared to healthy controls (HCs).

**Methods:**

Participants were exposed to immersive VR scenarios designed to elicit different awe experiences (mountains, waterfall, Earth) and a reference (awe-neutral) scenario. EEG activity was recorded during VR exposure and at baseline, followed by emotional state questionnaires. Power spectral density and graph-theoretical connectivity indices – Nodal Positive Strength and Global Efficiency – were computed across theta, alpha, and beta bands.

**Results:**

Healthy controls showed high awe responses in awe-inducing scenarios with selective, scenario-specific modulations in alpha and theta band activity and connectivity, reflecting preserved cognitive flexibility. Conversely, ADs reported similar awe responses across all VR scenarios with reduced environmental differentiation. With respect to HCs, ADs showed elevated theta power in bilateral frontal and temporal regions, suggesting compensatory activity related to emotional processing alterations. Both groups exhibited VR-induced reductions in alpha-band global efficiency, more pronounced in ADs, suggesting compromised neural integration during complex emotional processing.

**Discussion:**

Taken together, the results suggest that the emotional processing deficits inherent to ADs may limit the capacity to engage differentially with emotionally complex stimuli such as awe, while nonetheless providing initial evidence that VR-based awe exposure combined with neurophysiological recording represents a valuable approach for discriminating differential cerebral emotional responses in clinical populations. This proof-of-concept work warrants further investigation in larger cohorts to evaluate the therapeutic potential of awe-based interventions for affective disorders.

## 1 Introduction

Affective disorders (ADs), also known as mood disorders, represent a group of psychological conditions characterized by alterations in emotional regulation, frequently presenting as depressive manifestations (Sekhon and Gupta, 2023). Among depressive conditions, Major Depressive Disorder (MDD) and Bipolar Disorder (BD) are leading causes of disability worldwide, significantly impacting quality of life and increasing the risk of medical comorbidities (Grande et al., 2016; Lam et al., 2024; Yan et al., 2024). Beyond their core diagnostic criteria, ADs are characterized by profound alterations in the cognitive-affective domain, while MDD is characterized by negative bias in implicit emotional processing predominates, with enhanced reactivity to negative emotional cues and reduced accessibility to positive emotional stimuli (Zhang et al., 2016), BD presents a more complex pattern of emotion dysregulation characterized by extreme affective reactivity, difficulties in emotion regulation across all mood states, and impairments in facial emotion recognition and reward processing (Townsend and Altshuler, 2012; De Prisco et al., 2023).

The neurobiological underpinnings of these emotional processing deficits involve functional alterations within multiple brain networks. Electroencephalographic (EEG) studies in ADs consistently report abnormalities in oscillatory activity associated with emotional and motivational processing. A well-replicated finding is frontal alpha-band asymmetry, which has been interpreted as a marker of impaired positive emotion processing and a bias toward negative affective states (Harmon-Jones et al., 2008; Fernández-Palleiro et al., 2020). In addition to alpha-band abnormalities, converging evidence highlights disruptions in theta-band activity across ADs. Increased frontal and anterior midline theta power, commonly associated with dysfunctional anterior cingulate cortex activity, has been robustly linked to impaired emotion regulation and heightened ruminative or internally focused processing on depression (Jaworska et al., 2012; Arns et al., 2015). Furthermore, Functional Magnetic Resonance Imaging (fMRI) studies on MDD show reduced deactivation in the pregenual anterior cingulate cortex, ventromedial prefrontal cortex, and posterior cingulate cortex, alongside altered activity in the dorsolateral prefrontal cortex when compared to control subjects (Grimm et al., 2009; Victor et al., 2013). Moreover, reduced functional connectivity has been observed between the medial temporal gyrus (MTG) and the ventral caudate nucleus, as well as other regions, alongside increased connectivity between the superior temporal gyrus and precuneus, and angular gyrus (Yang et al., 2017). The persistent hyperactivity of the Default Mode Network (DMN) during transitions from resting state to externally oriented tasks contribute to cognitive vulnerability by promoting rumination and depleting resources needed for adaptive emotional processing (Wang et al., 2016). Similarly, BD shows disrupted DMN connectivity, particularly in the precuneus, medial prefrontal cortex, and posterior cingulate cortex (Öngür et al., 2010; Townsend and Altshuler, 2012; Zhao et al., 2024). Importantly, BD is also associated with decreased anticorrelation between the DMN and the salience network, potentially reflecting difficulties in switching between internally and externally directed attention (Zhao et al., 2024).

Treatment options for AD primarily target mood symptoms, with current first-line treatments being a result of a combined use of pharmacotherapy with psychotherapy to achieve symptomatic relief (Sekhon and Gupta, 2023). In MDD, typical approaches include antidepressants and cognitive behavioral therapy, while BD management requires mood stabilizers and atypical antipsychotics, often combined with psychoeducation and family-focused therapy (Fountoulakis et al., 2005; Shah et al., 2017). However, emotional processing impairments persist beyond mood symptom resolution and predict relapses (Roiser et al., 2012; Ruhe et al., 2019). Beyond standard treatments, experiential interventions, particularly those targeting emotional processing deficits, may be necessary to fully address the emotional processing deficits of ADs (Pos et al., 2003; Yen et al., 2024). Recent research began to explore the therapeutic potential of complex emotions induced in controlled laboratory settings. Of particular interest is the emotional experience of awe, studied as a possible adjunctive pathway to enhance positive affect, shift the focus away from the Self, and broaden attentional scope, which could benefit patients with depression (Chirico and Gaggioli, 2021; Monroy and Keltner, 2022).

Awe is a complex emotion arising from vast, overwhelming, and/or threatening stimuli (Keltner and Haidt, 2003) that exceeds one’s current mental schemas and necessitates cognitive accommodation (Pérez et al., 2023). This emotion has been defined as a self-transcendent phenomenon (e.g., Yaden et al., 2017) featuring two core features, that is, an increased sense of connectedness to others and the environment, and a diminished sense of self, termed “the small self” (Piff et al., 2015; Yaden et al., 2017; Cavallaro and Rivera, 2025). At the same time, awe itself can trigger profound self-transcendent experiences characterized by alterations in the perception of space, time, and self-concept, placing it at the edge between an emotional state and an altered state of consciousness (Chirico and Yaden, 2018; Abatista and Cova, 2023; Jiang et al., 2024; Zhao et al., 2026).

The self-transcendent properties of awe – shifting attentional focus from self to others and fostering a sense of connection to something greater – present compelling mechanisms for addressing core features of ADs. According to the Matryoshka model of self-transcendence (Chirico and Gaggioli, 2021), these properties directly counteract fundamental aspects of depressive psychopathology, including rumination and maladaptive self-referential thinking (Hendricks, 2018; Lopes et al., 2020; Chirico and Gaggioli, 2021; Monroy et al., 2025). Beyond its self-transcendent characteristics, awe enhances creative thinking (Chirico et al., 2018b; Zhang et al., 2021; Pizzolante et al., 2024) and promotes prosocial behavior (Piff et al., 2015; Li et al., 2024), cognitive and social domains notably impaired in ADs (Chirico and Yaden, 2018; Chirico et al., 2018b; Jiang and Sedikides, 2022). Furthermore, awe’s capacity to trigger profound restructuring of mental schemas and self-perception suggests potential for neuroplastic modifications of the inflexible cognitive biases that are characteristic of these disorders (Beck and Bredemeier, 2016; Price and Duman, 2020).

At the neural level, preliminary evidence from fMRI suggests that awe engages neural circuits relevant to the pathophysiology of ADs, supporting its potential therapeutic mechanisms. Neuroimaging research in healthy individuals has identified activation of the frontoparietal network, including medial frontal gyrus, insula, and supramarginal gyrus, as well as reduced DMN activity (van Elk et al., 2019). Additionally, the left MTG plays a pivotal role in processing both positive and threatening variants of awe (Takano and Nomura, 2022), while awe intensity appears modulated by the inferior temporal cortex, hippocampus, inferior and middle frontal gyri, basal ganglia, and cerebellum (Ishizu and Zeki, 2014).

However, while fMRI provides valuable insights into the brain hemodynamic correlates of awe with high spatial resolution, it presents inherent limitations for investigating neuronal dynamics, including oscillatory and connectivity patterns that unfold on millisecond timescales. Additionally, the fMRI environment constrains experimental paradigms to predominantly passive audio and/or visual stimuli. In this context, EEG offers complementary advantages for studying fast neuronal dynamics and connectivity patterns (Coelli et al., 2017, 2019; Maggioni et al., 2021) underlying awe experiences. Particularly, EEG provides direct measurement of brain electrical activity with exceptional temporal resolution, capturing the rapid neural dynamics that characterize emotional processing through quantification of oscillatory activity via power spectral measures and characterization of dynamic interactions between brain regions via connectivity metrics across the main frequency bands. Beyond its temporal advantages, EEG’s compatibility with diverse experimental settings broadens the methodological repertoire for awe induction. Among diverse awe-inducing technologies, virtual reality (VR) has emerged as a promising approach to combine experimental control and high ecological validity in the laboratory settings (Gallagher et al., 2015; Chirico et al., 2017; Hofmann et al., 2021; van Limpt-Broers et al., 2024). Immersive VR environments enable multisensorial stimulation and the creation of realistic scenarios (Chirico et al., 2016), thereby affording a unique sense of presence and immersion that enhances the emotional intensity of awe experiences.

Despite these methodological advantages, relatively few studies have investigated the electrophysiological correlates of awe in healthy individuals, especially those using VR paradigms. In our previous EEG-VR study, Carbone et al. (2024) investigated the brain mechanisms that underlie VR-induced awe experiences using different frequency-resolved metrics, among which spectral power and entropy-based measures. Within this work, three awe-inducing scenarios were compared with respect to a reference VR one. Across the awe-inducing scenarios, consistent metrics increases were found in selective channels spanning from the frontal and temporal lobes (theta, alpha, beta, gamma bands) to the parietal (beta band) and occipital (gamma band) lobes. Despite highly overlapping results, entropy-based metrics emerged to be particularly sensitive in capturing subtler scenario-specific changes, underscoring the importance of employing complementary EEG metrics to fully characterize brain processes associated with awe. Similarly, van Limpt-Broers et al. (2024) identified modulations in theta and beta band power during an awe-inducing VR experience simulating a rocket launch. Finally, Lee et al. (2025) characterized awe as an ambivalent affective state encompassing both positive and negative emotional components. These components were found to be associated with changes in frontoparietal multi-band oscillatory power.

The integration of EEG with immersive VR presents an unprecedented opportunity to investigate the neural dynamics of awe not only in healthy individuals, but also in clinical populations – such as those with ADs – who could benefit from awe-based experiential interventions. The ecological validity of VR-induced awe combined with the temporal precision of EEG can be used to explore whether experimentally induced awe experiences can modulate dysfunctional neural patterns that characterize emotional processing deficits in ADs. Such investigations are essential for determining whether awe-based interventions could serve as novel adjunctive treatments for the full spectrum of ADs.

In this study, for the first time to our knowledge, we aim to investigate the EEG activity and connectivity patterns underlying different awe experiences induced by validated VR scenarios (Chirico et al., 2018a) in a small sample of individuals with ADs with respect to a group of healthy controls (HCs).

## 2 Materials and methods

### 2.1 Participants

The sample consisted of 3 outpatients volunteers with ADs – 1 with BD (M, 24 years old) and 2 with MDD (F, 38 years old; F, 20 years old) – and 12 HCs (5 males, 7 females, 27.6 ± 5.0 years). All participants were between 20 and 40 years, had a normal or corrected vision, were not pregnant, and were capable of giving informed consent and complying with the study procedures. Individuals with ADs were diagnosed with affective disorders using the Italian version of the Structured Clinical Interview for DSM-5. Main exclusion criteria for HCs were meeting the DSM-5 criteria for psychotic, mood, or substance use disorders, either currently or in the past, balance or vestibular disorders, pregnancy, severe cognitive deficits, and intellectual disability. A more detailed list of exclusion criteria can be found in the registered protocol within the Open Science Framework (https://osf.io/m47du) and the relative study protocol paper (Bondi et al., 2025). Volunteers with ADs were enrolled from the Mood Disorders Day Hospital service in the Psychiatric Clinic of the Fondazione IRCCS Ca' Granda Ospedale Maggiore Policlinico, while controls were enrolled from the general Italian population. A written informed consent was obtained by all participants prior to the study. This study is compliant with the Helsinki Declaration and has been approved by the competent Ethical Committee on January 26th, 2021 (OSMAMI-26/01/2021-0002688-U).

### 2.2 Experimental design

The protocol included a resting-state EEG recording (baseline) and a subsequent VR session within which participants were asked to navigate in four different VR scenarios, three awe-inducing and one awe-neutral (also called reference), which were administered in a counterbalanced order. EEG data were recorded during navigation in each of the four VR scenarios.

The three awe-inducing scenarios, previously validated for awe elicitation by Chirico et al. (2018a), featured natural scenes of (1) a forest with tall trees (waterfall), (2) high mountains (mountains), and (3) the earth view from deep space (Earth). The reference scenario represents green grass with few flowers and trees and is used as control (awe-neutral) condition. The awe-inducing scenarios have been designed to feature a moment in which awe should reach its maximum intensity, hereinafter called peak of awe, which occurs when something unexpected happens after the exploration of a standardized navigation path (Bondi et al., 2025). During the EEG data acquisition of awe-inducing VR scenarios, the peak of awe was marked on the EEG as an event.

Beside neurophysiological measures, at baseline and after each VR scenario, the emotional status of participants was assessed using the Short Positive and Negative Affect Scale (PANAS) (Terracciano et al., 2006), and the Single Item Emotion (SIE) scale (Chirico et al., 2018a). Further details on the experimental protocol can be found in Bondi et al. (2025).

### 2.3 EEG data acquisition and pre-processing

At baseline and during each VR session, EEG data were continuously recorded using a Geodesic EEG system (GES 400, Electrical Geodesic Inc. (EGI), Philips, The Netherlands) equipped with a TMS-compatible 64-channel Geodesic EEG cap (MicroCel Geodesic Sensor Net 100, EGI, Philips, The Netherlands). The EEG sampling frequency was set to 1 kHz, and the reference electrode was placed at the vertex of the head.

The EEG data pre-processing was conducted using Matlab^®^ R2022a (Mathworks, Inc.), the EEGLAB toolbox (Delorme and Makeig, 2004), Matlab in-house scripts, and BrainVision Analyzer 2.0 R © software (Brain Products, Gilching, Germany). Sixty-second-long artifact-free segments were extracted for the baseline and reference VR scenarios, whereas a window of [−30:30] seconds around the peak of awe was used for the awe-inducing ones. The data were filtered using a finite impulse response band-pass filter (1–120 Hz) and a notch filter (50 Hz) to remove powerline noise and were down-sampled to 250 Hz. The BrainVision Analyzer software was then used to perform Independent Component Analysis (ICA) to detect and remove artifacts related to eye blinks, muscle movements, and single electrodes. The EEG signal was then reconstructed using the artifact-free ICs and re-referenced to the channels’ common average. The clean EEG data were divided into 2-s epochs, and for the awe-inducing VR scenarios, only the epochs after the awe peak were retained. Power spectral density (PSD) and functional connectivity (FC) were then computed across the theta, alpha, and beta bands for each epoch.

### 2.4 Emotional questionnaires

The emotional changes induced by the VR protocol relative to the baseline were evaluated computing the percentage-normalized scores for the PANAS and SIE questionnaires items for each VR scenario using the following formula:


$$\text{SCOR}E_{\mathrm{VR}\;\text{percentage}-\text{normalized}}=\frac{\left(\begin{array}{l} \text{SCOR}E_{\mathrm{VR}}-\\ \text{SCOR}E_{\text{baseline}} \end{array}\right)}{\text{SCOR}E_{\text{baseline}}}*100$$ (1)

Where VR is the scenario (i.e., reference, Earth, waterfall, and mountains), $\text{SCOR}E_{\mathrm{VR}\;\text{percentage}-\text{normalized}}$ is the percentage-normalized item score of the VR scenario, $\text{SCOR}E_{\mathrm{VR}}$ is the item score of the VR scenario, and $\text{SCOR}E_{\text{baseline}}$ refers to the item score of the questionnaire administered at baseline.

### 2.5 PSD and FC features

The power analysis was performed on individual EEG epochs using the Fast Fourier Transform method. Spectral power was quantified across three frequency bands: theta (4–8 Hz), alpha (8–13 Hz), and beta (13–30 Hz). For each band, power was computed through trapezoidal integration across the corresponding frequency range. Relative PSD (rPSD) values were obtained by dividing the power in each band by the total integrated power spanning 1 Hz to the Nyquist frequency. These rPSD values were then averaged across epochs for each participant, experimental condition, and frequency band. To quantify the changes induced by the VR compared to baseline levels, the percentage-normalized rPSD was derived via Equation 1.

The imaginary part of coherency (iCOH), which measures the brain functional connectivity by isolating true neural interactions by excluding zero-lag correlations arising from volume conduction artifacts (Nolte et al., 2004), was computed using the FieldTrip toolbox (Oostenveld et al., 2011). The EEG data underwent Fourier decomposition via multitaper methods with Hanning windows across the 1–120 Hz range. Similarly to the rPSD normalization approach, band-specific connectivity was determined through trapezoidal integration and normalized by total iCOH, in a manner similar to the rPSD normalization approach.

Graph-theoretic metrics were extracted using the Brain Connectivity Toolbox (Rubinov et al., 2009) in MATLAB. After normalizing the FC adjacency matrices and setting the diagonal elements to zero, node positive strength (NPS) and global efficiency (GE) were computed for each experimental condition and frequency band. NPS, the sum of weights of links connected to the node, reflects nodal centrality within the network. GE, calculated as the mean inverse shortest path length between all node pairs, with higher values indicating more direct communication pathways between nodes, was used as a measure of network integration. As with spectral analyses, connectivity metrics were expressed as normalized-percentage changes from baseline (Equation 1).

### 2.6 Statistical analysis

The Wilcoxon rank-sum test was used to evaluate differences in the percentage-normalized rPSD and graph-theory EEG measures between the ADs and HCs groups in each VR scenario and frequency band. The Wilcoxon signed-rank test was used to evaluate differences between awe-inducing VR scenarios and the reference one in each group and frequency band. Significance thresholds were set at *p* < 0.05 (uncorrected for multiple comparisons due to the exploratory nature of the study). The same statistical tests were performed for the PANAS and SIE percentage-normalized scores. Given the exploratory nature of this study and the constraints of the EEG analytical approach, *p*-values are reported for descriptive purposes only and should not be interpreted as confirmatory evidence.

## 3 Results

### 3.1 Emotional questionnaires: within- and between-group comparisons

The PANAS and SIE median scores for each group and condition are reported in Tables 1, 2. No differences (*p* < 0.05) were found for the PANAS scale, neither between groups nor conditions. Contrarily, differences within the HCs group and between ADs and HCs were found for the Amused, Happy, and Awe items of the SIE scale. HCs showed differences for the Amused item between reference and Earth (*p* < 0.05) and reference and waterfall (*p* < 0.01) scenarios; for the Happy item between reference and Earth (*p* < 0.05) and reference and waterfall (*p* < 0.05) scenarios; for the Awe item between reference and Earth (*p* < 0.01) and reference and mountains (*p* < 0.01) scenarios. Meanwhile, differences between groups were found for the Amused item in the waterfall scenario (*p* < 0.05); for the Happy item in the Earth (*p* < 0.05) and waterfall (*p* < 0.05) scenarios; for the Awe item in the reference (*p* < 0.05), waterfall (*p* < 0.05), and mountains (*p* < 0.05) scenarios.

**Table 1:** Median PANAS scores for healthy controls (HC) and affective disorders (AD).

	Baseline HC	Baseline AD	Reference HC	Reference AD	Earth HC	Earth AD	Waterfall HC	Waterfall AD	Mountain HC	Mountain AD
Enthusiastic	3	3	3	4	3	3	3	3	2,5	4
Determined	3,5	3	2,5	3	3	2	3,5	4	3	5
Attentive	4	4	3	4	3	1	3,5	4	3,5	5
Excited	3	2	2,5	4	3	3	3	2	2	3
Inspired	2,5	2	2	3	3	2	3	2	2	4
*Positive Affect*	16,5	13	14	18	14	11	15,5	14	12	21
Nervous	2	2	2	1	1,5	4	1	1	2	2
Afraid	1,5	1	1	2	1	1	1	1	1	1
Upset	2,5	1	1,5	1	1	2	1	1	1,5	2
Distressed	1	1	1	2	1	1	1	1	1	1
Scared	1	1	1	1	1	1	1	1	1	1
*Negative Affect*	8	6	6	7	5,5	9	5	5	7	7

**Table 2:** Median SIE scores for healthy controls (HC) and affective disorders (AD).

	Baseline HC	Baseline AD	Reference HC	Reference AD	Earth HC	Earth AD	Waterfall HC	Waterfall AD	Mountain HC	Mountain AD
Angry	1	1	1	2	1	6	1	1	1	1
Disgusted	1	1	1,5	1	1	3	1	1	1	1
Afraid	1	1	1	1	1	2	1	1	1	1
Proud	2	3	1	3	2	2	1,5	1	2	4
Amused	4	5	2	5	3,5	4	4	2	3,5	6
Sad	1	2	1	1	1	1	1	1	1	1
Happy	3,5	4	2,5	1	4,5	1	4	1	3	4
Awe	3	1	3	4	5	3	3,5	4	4,5	5

### 3.2 rPSD between-group comparison: VR scenarios

**Figure 1:**
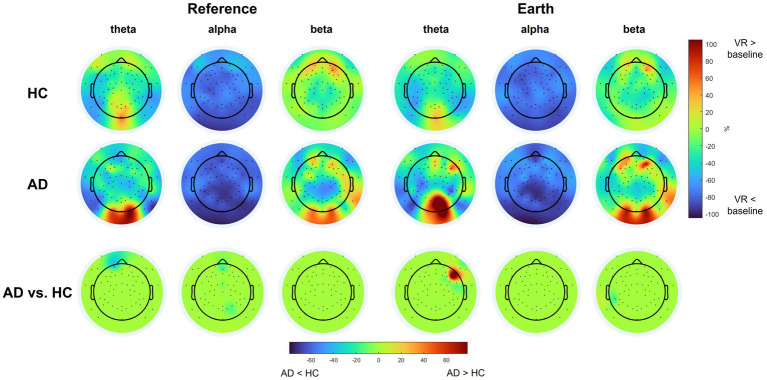
The relative power spectral density (rPSD) for each group [healthy controls (HC) and affective disorder (AD)] in the theta, alpha, and beta bands is shown for the reference and Earth scenarios. The topoplot values refer to the median percentage-normalized – with respect to baseline – value of rPSD for the VR scenarios. Difference in median percentage-normalized rPSD between ADs and HCs for electrodes showing group differences (Wilcoxon rank-sum test, *p* < 0.05).

**Figure 2:**
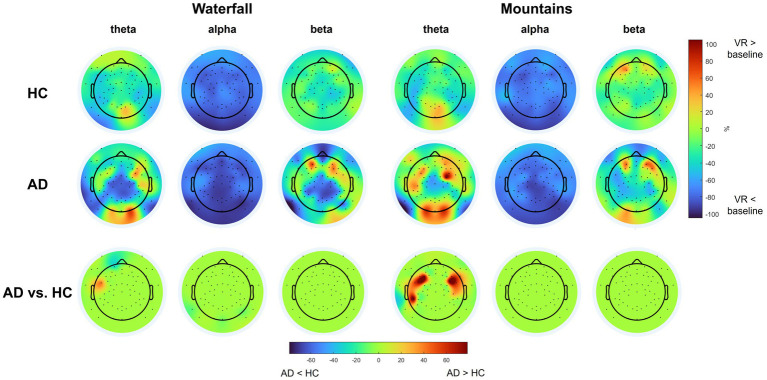
The relative power spectral density (rPSD) for each group [healthy controls (HC) and affective disorder (AD)] in the theta, alpha and beta bands is shown for the Waterfall and Mountains scenarios. The topoplot values refer to the median percentage-normalized – with respect to baseline – value of rPSD for the VR scenarios. Difference in median percentage-normalized rPSD between ADs and HCs for electrodes showing group differences (Wilcoxon rank-sum test, *p* < 0.05).

As shown in Figures 1, 2, when comparing the ADs and HCs groups, differences (*p* < 0.05) in terms of rPSD were mostly found in theta and alpha bands. Specifically, in the theta band the mountains scenario showed changes in the bilateral frontal (*p* < 0.01) and temporal areas, with an increase (with respect to baseline) for ADs and a decrease for HCs. While both groups exhibited negative rPSD in the alpha band in all VR scenarios, with respect to baseline, ADs showed lower values in the occipital regions in both the reference and waterfall scenarios, and in the frontal and frontocentral regions in the reference one only.

### 3.3 rPSD within-group comparison: awe-inducing vs. reference scenarios

**Figure 3:**
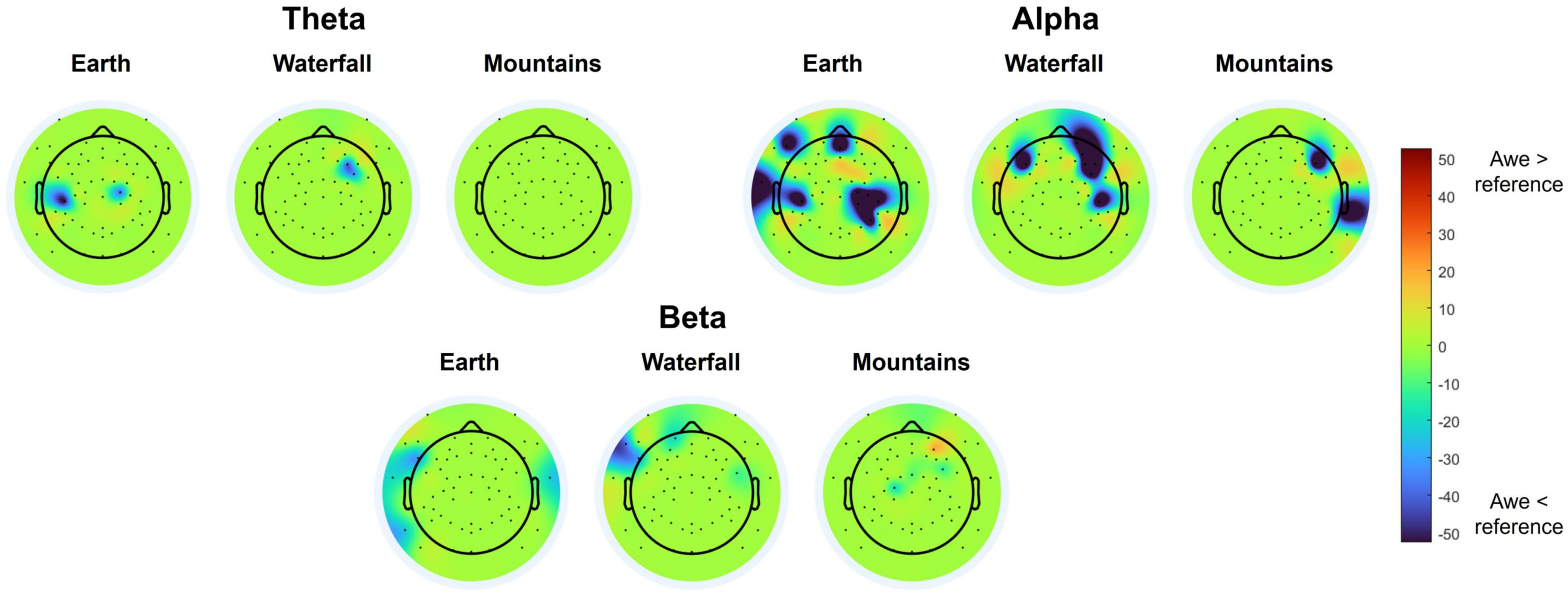
Difference in median percentage-normalized relative power spectral density (rPSD) between awe-inducing and reference scenarios for electrodes showing within-group differences in healthy controls (Wilcoxon signed-rank test, *p* < 0.05).

When comparing awe-inducing VR scenarios (mountains, Earth, and waterfalls) rPSD with the reference one, differences were observed in all frequency bands, solely in the HCs group (Figure 3). In the theta band, a reduction (*p* < 0.05) of rPSD is observed predominantly in the left temporal area in the Earth environment and the right frontal area in the waterfall environment. In the alpha band, lower rPSD values were observed bilaterally in the frontal and temporal regions, with the latter being more widespread in the Earth scenario. Finally, the beta band showed lower rPSD values in bilateral temporal regions for the Earth scenario, bilateral frontal for the Waterfall, and central regions for the mountains. A higher rPSD value was observed in the right frontal region for the mountains scenario.

### 3.4 NPS between-group comparison: VR scenarios

**Figure 4:**
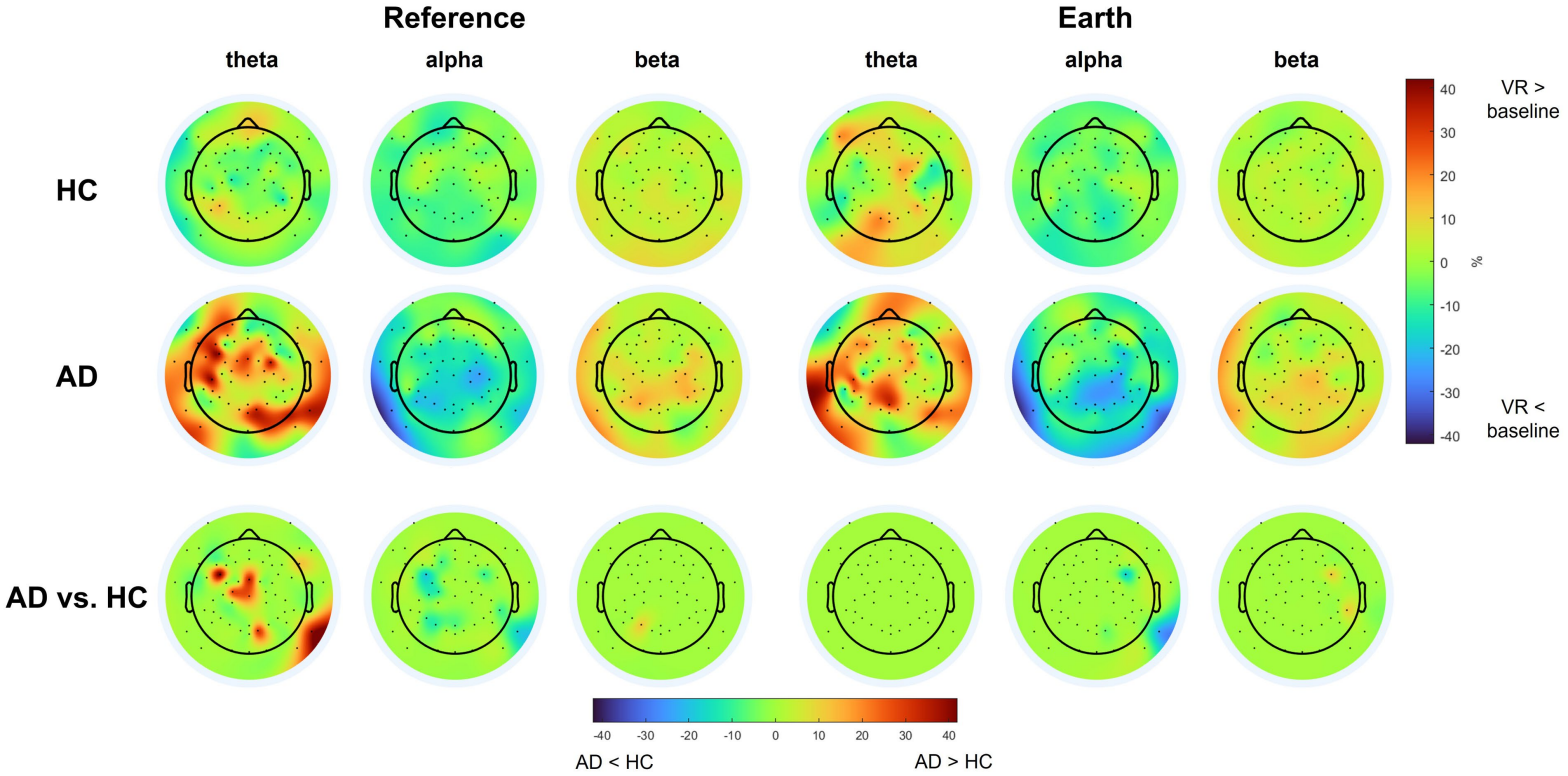
The node positive strength (NPS) for each group [healthy controls (HC) and affective disorder (AD)] in the theta, alpha, and beta bands is shown for the reference and Earth scenarios. The topoplot values refer to the median percentage-normalized – with respect to baseline – value of NPS for the VR scenarios. Difference in median percentage-normalized NPS between ADs and HCs for electrodes showing group differences (Wilcoxon rank-sum test, *p* < 0.05).

**Figure 5:**
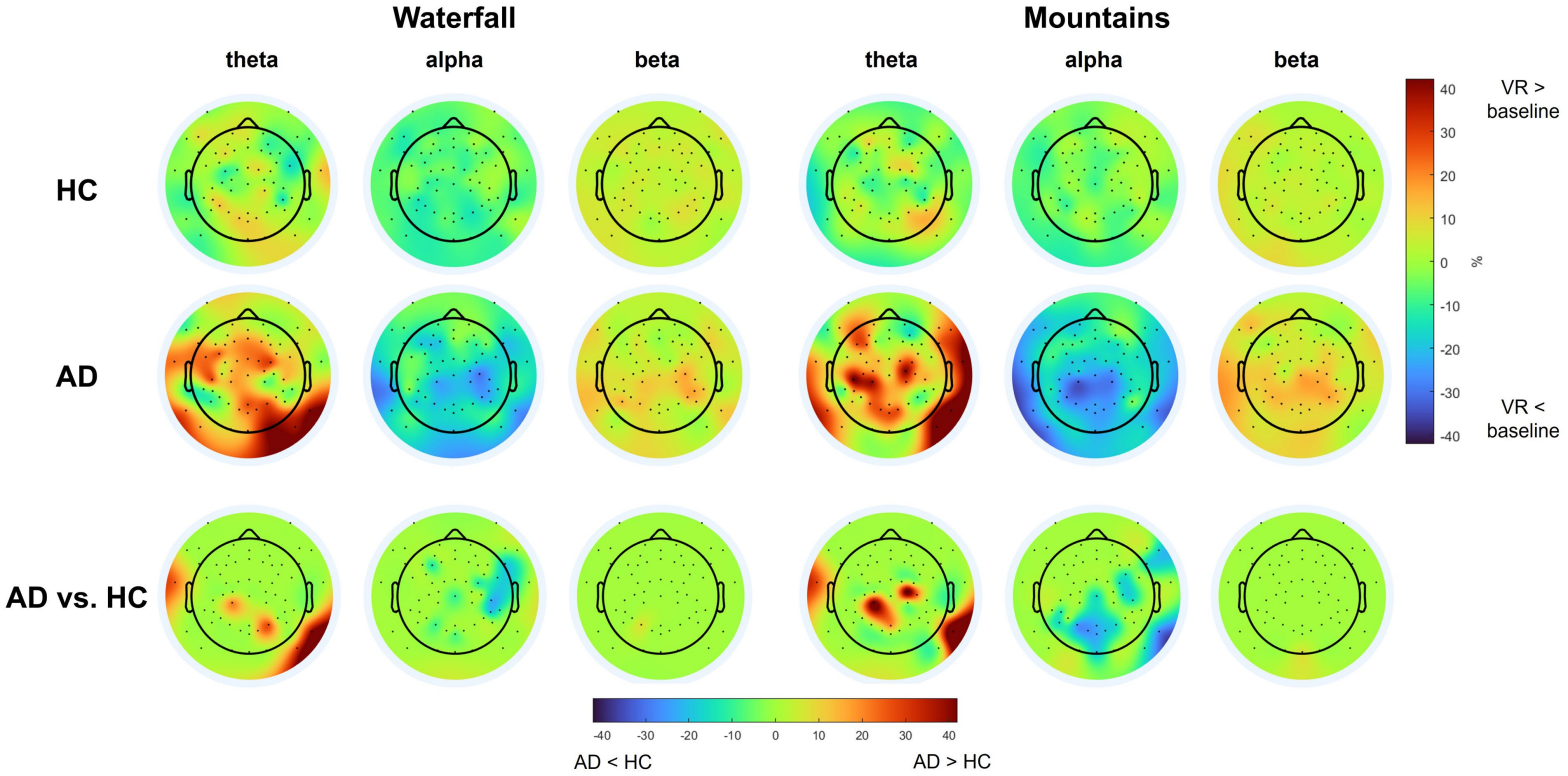
The relative node positive strength (NPS) for each group [healthy controls (HC) and affective disorder (AD)] in the theta, alpha and beta bands is shown for the Waterfall and Mountains scenarios. The topoplot values refer to the median percentage-normalized – with respect to baseline – value of NPS for the VR scenarios. Difference in median percentage-normalized NPS between ADs and HCs for electrodes showing group differences (Wilcoxon rank-sum test, *p* < 0.05).

As shown in Figures 4, 5, theta-band NPS increased in ADs with respect to controls (*p* < 0.05), with a widespread bilateral distribution involving frontal, paramedial and centro-parietal areas, the latter of which were predominantly engaged during the reference, waterfall, and mountains scenarios. The results for the alpha band were substantially similar, with the same regions showing a reduction in NPS in both groups. However, these changes were more pronounced in the ADs group than in the HCs group in the paramedial and parietal areas (reference), the right frontal and temporal regions (Earth), the right temporal and centro-parietal regions (waterfall) and the central and temporal regions (mountains). Lastly, in beta-band, ADs showed higher NPS in the parietal left region for the reference and waterfall scenarios, left tempo-frontal region for Earth, and occipital for the mountains scenario.

### 3.5 NPS within-group comparison: awe-inducing vs. reference scenarios

**Figure 6:**
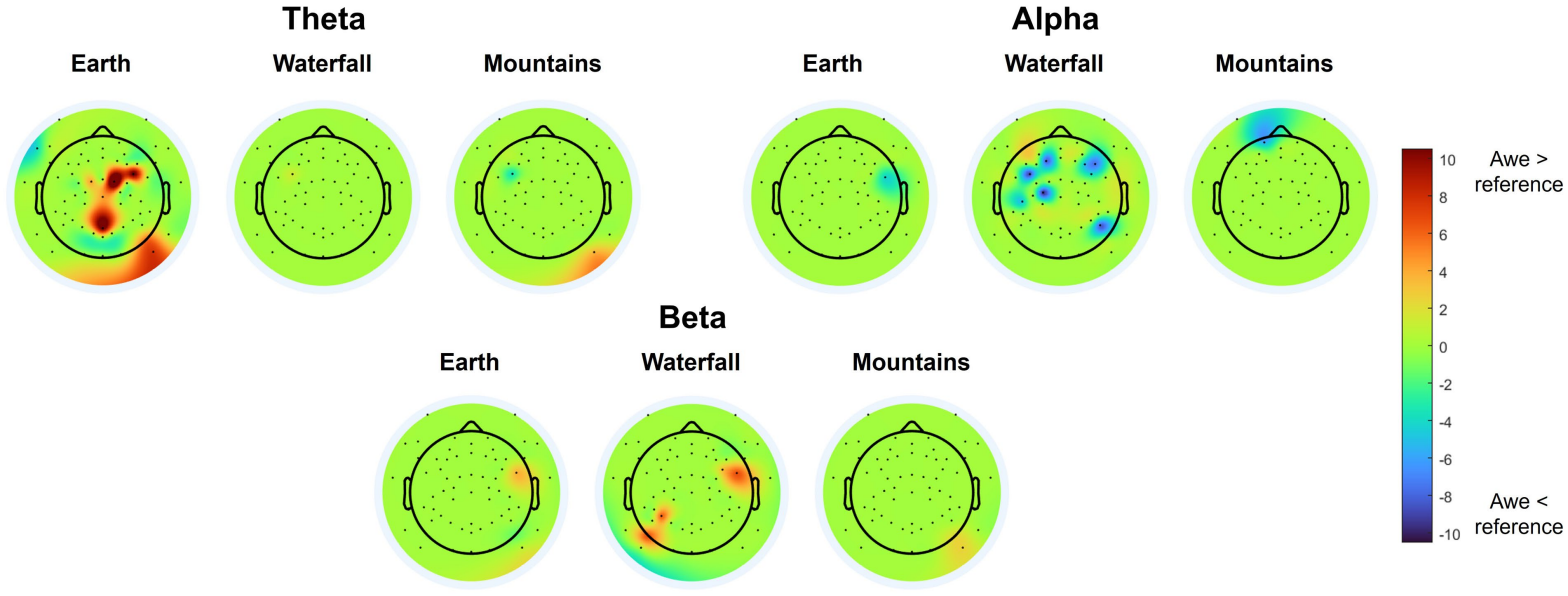
Difference in median node postive strength (NPS) between awe-inducing and reference scenarios for electrodes showing within-group differences in healthy controls (Wilcoxon signed-rank test, *p* < 0.05).

Variations in NPS (p < 0.05), between awe-inducing and reference scenarios, emerged exclusively within the HCs group (Figure 6). Particularly, a marked increase in NPS was detected across frontal and central areas and in the right temporo-occipital region for the Earth scenario, within the theta band. By contrast, NPS reduction in the alpha frequency band was predominantly observed in the fronto-temporal regions, predominantly for the waterfall scenario. The same scenario showed a fronto-temporal NPS increase in the beta band.

### 3.6 GE between-group comparison: VR scenarios

The GE results showed greater values for the ADs group and consistent alpha-band negative values for both groups across all VR scenarios (Table 3). Differences in alpha band GE were found between the ADs and HCs groups (*p* < 0.05), with the ADs group showing lower GE values than the HCs group in the waterfall (−14.26 vs. −6.61, *p* < 0.05) and mountains (−15.16 vs. −2.71, *p* < 0.05) scenarios.

**Table 3:** Percentage-normalized Global Efficiency (GE) values for theta, alpha, and beta band in virtual reality (VR) scenarios for healthy controls (HC) and affective disorders (AD).

	Reference HC	Reference AD	Earth HC	Earth AD	Waterfall HC	Waterfall AD	Mountain HC	Mountain AD
Theta	−3.06	11.14	5.26	7.35	1.42	10.61	2.18	16.05
Alpha	−6.82	−15.08	−7.36	−10.74	−6.61*	−14.26*	−2.71*	−15.16*
Beta	2.73	4.86	0.39	5.74	4.22	5.87	3.83	5.36

*Wilcoxon rank-sum test (*p* < 0.05).

### 3.7 GE within-group comparison: awe-inducing vs. reference scenarios

No differences in GE (*p* < 0.05) were observed for both groups when the three awe-inducing conditions were compared to the reference one.

## 4 Discussion

In this study, for the first time to our knowledge, the EEG neural correlates underlying awe-inducing VR experiences were investigated in a small cohort of individuals with ADs compared to HCs. Despite the exploratory nature of this work, our results allowed us to capture differences between groups in terms of both emotional and EEG correlates, also suggesting the differential effect induced by each awe-inducing scenario in both HCs and ADs.

In line with previous literature (Chirico et al., 2018a), our results suggest that healthy participants experienced higher intensity of awe (awe-item of the SIE scale) within the awe-inducing scenarios with respect to reference one, with the highest values following the navigation in the Earth environment, possibly reflecting lower familiarity with this setting. This trend differed in the ADs group, where all VR experiences induced awe, including the reference one, with the highest values recorded for the mountains scenario and the lowest for the Earth one. Moreover, differences between groups in terms of awe were associated with ADs group’ higher reported positive feelings (high PANAS positive affect scores) for the reference and mountain scenarios, and higher reported negative feelings (high PANAS negative affect scores) for the Earth scenario. These exploratory results seem to suggest a positive effect of immersive scenarios in the patient group, except for the Earth scenario, with limited differences among reference and awe-inducing scenarios.

In terms of EEG power activity, our study revealed a between-group difference in theta-band PSD activity in bilateral frontal and temporal areas, which was enhanced in ADs compared to HCs, specifically during the mountains scenario. Interestingly, these cortical regions have been associated with the elaboration of awe experiences (Takano and Nomura, 2022), whereas frontal theta band activity has been associated with emotional and behavioral control in the anterior cingulate cortex (Fernández-Palleiro et al., 2020). Hence, within our experimental framework, this theta-band enhancement may suggest compensatory activity related to emotional processing alterations in ADs.

In terms of connectivity, our results reveal distinct between-group differences across alpha and theta bands, which appear to reflect both the physiological underpinnings of awe processing and the emotional dysregulation characteristic of ADs. In the alpha band, the reduction of NPS and GE observed in VR environments – particularly during the awe-inducing waterfall and mountains scenarios relative to baseline – was broader and more widespread in ADs, with marked involvement of frontal, paramedial, and especially temporal and parietal regions. This seems to suggest a loss of the typical alpha-band lateralization that characterizes emotional processing in healthy individuals, further compromising an already dysfunctional pattern in ADs (Jaworska et al., 2012; Fernández-Palleiro et al., 2020). In HCs, if transient, such a reduction may be interpreted as physiological, insofar as the experience of awe promotes inward perceptual-emotional processes of a contemplative nature through the strengthening of connections within the DMN, at the expense of global integration (Aftanas and Golocheikine, 2001; Cahn and Polich, 2006). However, in individuals with ADs, this awe-induced reduction in GE may become accentuated and potentially less reversible, suggesting that these individuals are more vulnerable in maintaining the balance between segregation and interhemispheric integration of neural information, ultimately resulting in impaired affective regulation during complex emotional states (Bush et al., 2000; Etkin et al., 2011). In the theta band, HCs exhibited a decrease in NPS in fronto-central regions during the reference scenario, whereas an opposite trend emerged in the awe-inducing scenarios, particularly in the medial and paramedial regions of the Earth scenario. This pattern may reflect a “contemplative” state physiologically induced by the awe experience (Aftanas and Golocheikine, 2001; Baijal and Srinivasan, 2010), potentially facilitating interhemispheric connectivity – in which ACC activation is thought to play a pivotal role (Ishii et al., 1999; Nishida et al., 2004; Etkin et al., 2011) – and thereby enhancing emotional regulation and cognitive flexibility (Cavanagh and Shackman, 2014). Conversely, ADs showed a generalized increase in theta-band NPS across all VR scenarios, with higher values in fronto-central medial regions for the reference scenario and in central and parietal regions for the waterfall and mountains scenarios, hence possibly reflecting a dysregulation of emotional processes (Pizzagalli, 2011).

A key finding emerging from both activity and connectivity analyses is the differential sensitivity to environmental context between groups. In HCs, a selective modulation of neural responses was observed as a function of both the presence or absence of awe and the specific nature of each awe-inducing scenario, across alpha and theta bands. This may indicate a preserved ability to adapt flexibly to stimuli of diverse nature, with a greater degree of neural modulation reflecting intact salience processing (Bressler and Menon, 2010). By contrast, individuals with ADs exhibited a largely uniform neural response across different environments, regardless of whether awe was elicited. This pattern suggests that the emotional processing deficits inherent to ADs are linked to an alteration in salience mechanisms – i.e., the attribution of significance to events and experiences (Seeley et al., 2007; Menon, 2011) – making these individuals more susceptible to misattribution of environmental stimuli. Indeed, altered salience in ADs leads to a heightened attentional bias toward negatively valenced stimuli, with a consequent misrecognition of positive or neutral ones, and more broadly, to difficulty in shifting from a self-referential state to one of environmental exploration (Guha et al., 2021; Willinger et al., 2024). It may further be assumed that this process becomes even more complex when processing a multifaceted emotion such as awe, which encompasses both positive and negative valence and is therefore characterized by ambivalent nature.

Finally, our results pointed to differential activation in terms of both EEG power and local and global connectivity strength especially in the theta band, accompanied by a difficulty in differentiating between reference and awe-inducing VR scenarios in the patient group, possibly reflecting the dysregulation of emotional processing typical of ADs individuals and alteration in salience mechanisms. However, several limitations should be acknowledged. Due to the exploratory nature of the study, the sample was relatively small and unevenly distributed between the two groups, preventing robust statistical analysis and correlations between EEG measures and clinical variables. Caution should be exercised when generalizing or transferring these findings to other contexts or similar patient populations. Nevertheless, this study suggests the capability of recording EEG during VR-based awe exposure in clinical settings and that the combination of VR exposure techniques with neurophysiological recording methods may represent a valuable approach for discriminating against differential cerebral emotional responses between individuals with ADs and healthy individuals. Future studies could more precisely characterize clinical features, emphasizing symptom severity and including additional domains such as cognition. This proof-of-concept work provides a foundation for testing the specific hypotheses that emerged from our findings in larger, more controlled investigations, and represents a first step toward a deeper understanding of awe experience and its therapeutic potentialities across different psychiatric disorders through repeated experimental sessions.

## Data Availability

The datasets presented in this article are not readily available because of the sensitive nature of the research. Data are available for researchers who meet the criteria for access to confidential data. Requests to access the datasets should be directed to the corresponding author, Paolo Brambilla, paolo.brambilla1@unimi.it.

## References

[ref1] Abatista A. G. F. Cova F. (2023). Are self-transcendent emotions one big family? An empirical taxonomy of positive self-transcendent emotion labels. Affect. Sci. 4, 731–743. doi: 10.1007/S42761-023-00194-1 38156249 PMC10751273

[ref2] Aftanas L. I. Golocheikine S. A. (2001). Human anterior and frontal midline theta and lower alpha reflect emotionally positive state and internalized attention: high-resolution EEG investigation of meditation. Neurosci. Lett. 310, 57–60. doi: 10.1016/S0304-3940(01)02094-8, 11524157

[ref3] Arns M. Etkin A. Hegerl U. Williams L. M. DeBattista C. Palmer D. M. . (2015). Frontal and rostral anterior cingulate (rACC) theta EEG in depression: implications for treatment outcome? Eur. Neuropsychopharmacol. 25, 1190–1200. doi: 10.1016/j.euroneuro.2015.03.007 25936227

[ref4] Baijal S. Srinivasan N. (2010). Theta activity and meditative states: spectral changes during concentrative meditation. Cogn. Process. 11, 31–38. doi: 10.1007/S10339-009-0272-0, 19626355

[ref5] Beck A. Bredemeier K. (2016). A unified model of depression: integrating clinical, cognitive, biological, and evolutionary perspectives. Clin. Psychol. Sci. 4, 596–619. doi: 10.1177/2167702616628523

[ref6] Bondi E. Carbone F. Pizzolante M. Schiena G. Ferro A. Mazzocut-Mis M. . (2025). Integrating virtual reality, electroencephalography, and transcranial magnetic stimulation to study the neural correlates of awe experiences: the SUBRAIN protocol. PLoS One 20, 1–16. doi: 10.1371/journal.pone.0302762 PMC11964456 40173407

[ref800] Bressler S. L. Menon V. (2010). Large-scale brain networks in cognition: emerging methods and principles. Trends Cogn. Sci. 114, 277–290. doi: 10.1016/j.tics.2010.04.004, 20493761

[ref7] Bush G. Luu P. Posner M. I. (2000). Cognitive and emotional influences in anterior cingulate cortex. Trends Cogn. Sci. 4, 215–222. doi: 10.1016/S1364-6613(00)01483-2 10827444

[ref8] Cahn B. R. Polich J. (2006). Meditation states and traits: EEG, ERP, and neuroimaging studies. Psychol. Bull. 132, 180–211. doi: 10.1037/0033-2909.132.2.180, 16536641

[ref9] Carbone F. Bondi E. Massalha Y. Anastasi A. Ferro A. Pizzolante M. . (2024). Exploring brain activity during awe-inducing virtual reality experiences: a multi-metric EEG frequency analysis. Proceedings of the Annual International Conference of the IEEE Engineering in Medicine and Biology Society, EMBS. 10.1109/EMBC53108.2024.10782046 40039568

[ref10] Cavallaro R. M. Rivera G. N. (2025). Feeling small but still connected: examining complex effects of awe on self-compassion. Curr. Psychol. 44, 9846–9864. doi: 10.1007/S12144-025-07706-1

[ref11] Cavanagh J. F. Shackman A. J. (2014). Frontal midline theta reflects anxiety and cognitive control: Meta-analytic evidence. J. Physiol. Paris 109, 3–15. doi: 10.1016/j.jphysparis.2014.04.003, 24787485 PMC4213310

[ref12] Chirico A. Cipresso P. Yaden D. B. Biassoni F. Riva G. Gaggioli A. (2017). Effectiveness of immersive videos in inducing awe: an experimental study. Sci. Rep. 7:1218. doi: 10.1038/s41598-017-01242-0, 28450730 PMC5430774

[ref13] Chirico A. Ferrise F. Cordella L. Gaggioli A. (2018a). Designing awe in virtual reality: an experimental study. Front. Psychol. 8:293522. doi: 10.3389/fpsyg.2017.02351 PMC5786556 29403409

[ref14] Chirico A. Gaggioli A. (2021). The potential role of awe for depression: reassembling the puzzle. Front. Psychol. 12:617715. doi: 10.3389/fpsyg.2021.617715, 33981268 PMC8107378

[ref15] Chirico A. Glaveanu V. P. Cipresso P. Riva G. Gaggioli A. (2018b). Awe enhances creative thinking: an experimental study. Creat. Res. J. 30, 123–131. doi: 10.1080/10400419.2018.1446491

[ref16] Chirico A. Yaden D. B. (2018). “Awe: a self-transcendent and sometimes transformative emotion,” in The Function of Emotions: When and Why Emotions Help Us, Cham: Springer International Publishing, 221–233. doi: 10.1007/978-3-319-77619-4_11

[ref17] Chirico A. Yaden D. B. Riva G. Gaggioli A. (2016). The potential of virtual reality for the investigation of awe. Front. Psychol. 7:223153. doi: 10.3389/fpsyg.2016.01766, 27881970 PMC5101419

[ref18] Coelli S. Maggioni E. Cerutti S. Nobili L. Rubino A. Campana C. . (2017). Functional clustering approach for the analysis of stereo-EEG activity patterns in correspondence of epileptic seizures. Proceedings of the Annual International Conference of the IEEE Engineering in Medicine and Biology Society EMBS, 2806–2809 10.1109/EMBC.2017.8037440 29060481

[ref19] Coelli S. Maggioni E. Rubino A. Campana C. Nobili L. Bianchi A. M. (2019). Multiscale functional clustering reveals frequency dependent brain Organization in Type II focal cortical dysplasia with sleep Hypermotor epilepsy. I.E.E.E. Trans. Biomed. Eng. 66, 2831–2839. doi: 10.1109/TBME.2019.2896893, 30716026

[ref20] De Prisco M. Oliva V. Fico G. Radua J. Grande I. Roberto N. . (2023). Emotion dysregulation in bipolar disorder compared to other mental illnesses: a systematic review and meta-analysis. Psychol. Med. 53, 7484–7503. doi: 10.1017/S003329172300243X, 37842774 PMC10951413

[ref21] Delorme A. Makeig S. (2004). EEGLAB: an open source toolbox for analysis of single-trial EEG dynamics including independent component analysis. J. Neurosci. Methods 134, 9–21. doi: 10.1016/j.jneumeth.2003.10.009, 15102499

[ref22] Etkin A. Egner T. Kalisch R. (2011). Emotional processing in anterior cingulate and medial prefrontal cortex. Trends Cogn. Sci. 15, 85–93. doi: 10.1016/J.TICS.2010.11.004, 21167765 PMC3035157

[ref23] Fernández-Palleiro P. Rivera-Baltanás T. Rodrigues-Amorim D. Fernández-Gil S. Vallejo-Curto M. C. Álvarez-Ariza M. . (2020). Brainwaves oscillations as a potential biomarker for major depression disorder risk. Clin. EEG Neurosci. 51, 3–9. doi: 10.1177/1550059419876807, 31537100

[ref470] Fountoulakis K. N. Vieta E. Sanchez-Moreno J. Kaprinis S. G. Goikolea J. M. Kaprinis G. S. . (2005). Treatment guidelines for bipolar disorder: a critical review. J. Affect. Disord. 86, 1–10. 10.1016/j.jad.2005.01.004 15820265

[ref24] Gallagher S. Reinerman-Jones L. Janz B. Bockelman P. Trempler J. (2015). A Neurophenomenology of Awe and Wonder. New York: Palgrave Macmillan.

[ref25] Grande I. Berk M. Birmaher B. Vieta E. (2016). Bipolar disorder. Lancet 387, 1561–1572. doi: 10.1016/S0140-6736(15)00241-X 26388529

[ref26] Grimm S. Boesiger P. Beck J. Schuepbach D. Bermpohl F. Walter M. . (2009). Altered negative BOLD responses in the default-mode network during emotion processing in depressed subjects. Neuropsychopharmacology 34, 932–943. doi: 10.1038/NPP.2008.81, 18536699

[ref27] Guha A. Yee C. M. Heller W. Miller G. A. (2021). Alterations in the default mode-salience network circuit provide a potential mechanism supporting negativity bias in depression. Psychophysiology 58:e13918. doi: 10.1111/psyp.13918, 34403515

[ref28] Harmon-Jones E. Abramson L. Y. Nusslock R. Sigelman J. D. Urosevic S. Turonie L. D. . (2008). Effect of bipolar disorder on left frontal cortical responses to goals differing in valence and task difficulty. Biol. Psychiatry 63, 693–698. doi: 10.1016/j.biopsych.2007.08.004, 17919457

[ref29] Hendricks P. S. (2018). Awe: a putative mechanism underlying the effects of classic psychedelic-assisted psychotherapy. Int. Rev. Psychiatry 30, 331–342. doi: 10.1080/09540261.2018.1474185 30260256

[ref30] Hofmann S. M. Klotzsche F. Mariola A. Nikulin V. V. Villringer A. Gaebler M. (2021). Decoding subjective emotional arousal from EEG during an immersive virtual reality experience. eLife 10. doi: 10.7554/eLife.64812, 34708689 PMC8673835

[ref31] Ishii R. Shinosaki K. Ukai S. Inouye T. Ishihara T. Yoshimine T. . (1999). Medial prefrontal cortex generates frontal midline theta rhythm. Neuroreport 10, 675–679. doi: 10.1097/00001756-199903170-00003, 10208529

[ref32] Ishizu T. Zeki S. (2014). A neurobiological enquiry into the origins of our experience of the sublime and beautiful. Front. Hum. Neurosci. 8:112528. doi: 10.3389/fnhum.2014.00891, 25426046 PMC4227571

[ref33] Jaworska N. Blier P. Fusee W. Knott V. (2012). Alpha power, alpha asymmetry and anterior cingulate cortex activity in depressed males and females. J. Psychiatr. Res. 46, 1483–1491. doi: 10.1016/j.jpsychires.2012.08.003, 22939462 PMC3463760

[ref34] Jiang T. Hicks J. A. Yuan W. Yin Y. Needy L. Vess M. (2024). The unique nature and psychosocial implications of awe. Nat. Rev. Psychol. 3, 475–488. doi: 10.1038/s44159-024-00322-z

[ref35] Jiang T. Sedikides C. (2022). Awe motivates authentic-self pursuit via self-transcendence: implications for prosociality. J. Pers. Soc. Psychol. 123, 576–596. doi: 10.1037/pspi0000381, 34855435

[ref36] Keltner D. Haidt J. (2003). Approaching awe, a moral, spiritual, and aesthetic emotion. Cogn. Emot. 17, 297–314. doi: 10.1080/02699930302297, 29715721

[ref37] Lam R. W. Kennedy S. H. Adams C. Bahji A. Beaulieu S. Bhat V. . (2024). Canadian network for mood and anxiety treatments (CANMAT) 2023 update on clinical guidelines for management of major depressive disorder in adults. Can. J. Psychiatr. 69, 641–687. doi: 10.1177/07067437241245384, 38711351 PMC11351064

[ref38] Lee J. Han D. D. Oh S.-Y. Cha J. (2025). Awe is characterized as an ambivalent affect in the human behavior and cortex. Commun. Psychol. 3:123. doi: 10.1038/s44271-025-00299-2, 40804343 PMC12350678

[ref39] Li R. Hou Z. Zhang C. Xu Q. Nie A. (2024). A meta-analysis examining the relationship between awe and prosocial behavior. Curr. Psychol. 43, 24702–24711. doi: 10.1007/S12144-024-06039-9

[ref40] Lopes S. Lima M. Silva K. (2020). Nature can get it out of your mind: the rumination reducing effects of contact with nature and the mediating role of awe and mood. J. Environ. Psychol. 71:101489. doi: 10.1016/j.jenvp.2020.101489

[ref41] Maggioni E. Arienti F. Minella S. Mameli F. Borellini L. Nigro M. . (2021). Effective connectivity during rest and music listening: an EEG study on Parkinson’s disease. Front. Aging Neurosci. 13:657221. doi: 10.3389/fnagi.2021.657221, 33994997 PMC8113619

[ref42] Menon V. (2011). Large-scale brain networks and psychopathology: a unifying triple network model. Trends Cogn. Sci. 15, 483–506. doi: 10.1016/j.tics.2011.08.003, 21908230

[ref43] Monroy M. Amster M. Eagle J. Zerwas F. K. Keltner D. López J. E. (2025). Awe reduces depressive symptoms and improves well-being in a randomized-controlled clinical trial. Sci. Rep. 15:16453. doi: 10.1038/s41598-025-96555-w 40355653 PMC12069556

[ref44] Monroy M. Keltner D. (2022). Awe as a pathway to mental and physical health. Perspect. Psychol. Sci. 18, 309–320. doi: 10.1177/17456916221094856, 35994778 PMC10018061

[ref45] Nishida M. Hirai N. Miwakeichi F. Maehara T. Kawai K. Shimizu H. . (2004). Theta oscillation in the human anterior cingulate cortex during all-night sleep: an electrocorticographic study. Neurosci. Res. 50, 331–341. doi: 10.1016/j.neures.2004.08.004 15488296

[ref46] Nolte G. Bai O. Wheaton L. Mari Z. Vorbach S. Hallett M. (2004). Identifying true brain interaction from EEG data using the imaginary part of coherency. Clin. Neurophysiol. 115, 2292–2307. doi: 10.1016/j.clinph.2004.04.029, 15351371

[ref47] Öngür D. Lundy M. Greenhouse I. Shinn A. K. Menon V. Cohen B. M. . (2010). Default Mode Network Abnormalities in Bipolar Disorder and Schizophrenia. Psychiatry Res. 183, 59–68. doi: 10.1016/J.PSCYCHRESNS.2010.04.008 20553873 PMC2902695

[ref48] Oostenveld R. Fries P. Maris E. Schoffelen J. M. (2011). FieldTrip: open source software for advanced analysis of MEG, EEG, and invasive electrophysiological data. Comput. Intell. Neurosci. 2011, 1–9. doi: 10.1155/2011/156869, 21253357 PMC3021840

[ref49] Pérez K. A. Lench H. C. Thompson C. G. North S. (2023). Experimental elicitations of awe: a meta-analysis. Cogn. Emot. 37, 18–33. doi: 10.1080/02699931.2022.2140126, 36331080

[ref50] Piff P. K. Dietze P. Feinberg M. Stancato D. M. Keltner D. (2015). Awe, the small self, and prosocial behavior. J. Pers. Soc. Psychol. 108, 883–899. doi: 10.1037/PSPI0000018, 25984788

[ref290] Pizzagalli D. A. (2011). Frontocingulate dysfunction in depression: toward biomarkers of treatment response. Neuropsychopharmacology: official publication of the American College of Neuropsychopharmacology, 36, 183–206. doi: 10.1038/npp.2010.166 PMC3036952 20861828

[ref51] Pizzolante M. Pelowski M. Demmer T. R. Bartolotta S. Sarcinella E. D. Gaggioli A. . (2024). Aesthetic experiences and their transformative power: a systematic review. Front. Psychol. 15:1328449. doi: 10.3389/FPSYG.2024.1328449/BIBTEX, 39421842 PMC11484404

[ref52] Pos A. E. Greenberg L. S. Goldman R. N. Korman L. M. (2003). Emotional processing during experiential treatment of depression. J. Consult. Clin. Psychol. 71, 1007–1016. doi: 10.1037/0022-006X.71.6.1007 14622076

[ref53] Price R. B. Duman R. (2020). Neuroplasticity in cognitive and psychological mechanisms of depression: an integrative model. Mol. Psychiatry 25, 530–543. doi: 10.1038/s41380-019-0615-x, 31801966 PMC7047599

[ref530] Sekhon S. Gupta V. (2023). Mood disorder. FL: StatPearls Publishing., 32644337

[ref540] Shah N. Grover S. Rao G. P. (2017). Clinical practice guidelines for management of bipolar disorder. Indian J. Psychiatry. 59, S51–S66., 28216785 10.4103/0019-5545.196974 PMC5310104

[ref54] Roiser J. P. Elliott R. Sahakian B. J. (2012). Cognitive mechanisms of treatment in depression. Neuropsychopharmacology 37, 117–136. doi: 10.1038/npp.2011.183, 21976044 PMC3238070

[ref55] Rubinov M. Kötter R. Hagmann P. Sporns O. (2009). Brain connectivity toolbox: a collection of complex network measurements and brain connectivity datasets. NeuroImage 47:S169. doi: 10.1016/S1053-8119(09)71822-1

[ref56] Ruhe H. G. Mocking R. J. T. Figueroa C. A. Seeverens P. W. J. Ikani N. Tyborowska A. . (2019). Emotional biases and recurrence in major depressive disorder: results of 2.5 years follow-up of drug-free cohort vulnerable for recurrence. Front. Psych. 10:145. doi: 10.3389/fpsyt.2019.00145 PMC6447719 30984039

[ref57] Seeley W. W. Menon V. Schatzberg A. F. Keller J. Glover G. H. Kenna H. . (2007). Dissociable intrinsic connectivity networks for salience processing and executive control. J. Neurosci. 27, 2349–2356. doi: 10.1523/JNEUROSCI.5587-06.2007 17329432 PMC2680293

[ref58] Takano R. Nomura M. (2022). Neural representations of awe: distinguishing common and distinct neural mechanisms. Emotion 22, 669–677. doi: 10.1037/emo0000771, 32496077

[ref59] Terracciano A. McCrae R. R. Costa P. T. (2006). Factorial and construct validity of the Italian positive and negative affect schedule (PANAS). Psychol. Assess. 19, 131–141. doi: 10.1027/1015-5759.19.2.131 PMC2868265 20467578

[ref60] Townsend J. Altshuler L. L. (2012). Emotion processing and regulation in bipolar disorder: a review. Bipolar Disord. 14, 326–339. doi: 10.1111/J.1399-5618.2012.01021.X, 22631618

[ref61] van Elk M. Arciniegas Gomez M. A. van der Zwaag W. van Schie H. T. Sauter D. (2019). The neural correlates of the awe experience: reduced default mode network activity during feelings of awe. Hum. Brain Mapp. 40, 3561–3574. doi: 10.1002/hbm.24616, 31062899 PMC6766853

[ref62] van Limpt-Broers H. A. T. Postma M. van Weelden E. Pratesi S. Louwerse M. M. (2024). Neurophysiological evidence for the overview effect: a virtual reality journey into space. Virtual Real. 28, 1–19. doi: 10.1007/s10055-024-01035-7

[ref63] Victor T. A. Furey M. L. Fromm S. J. Öhman A. Drevets W. C. (2013). Changes in the neural correlates of implicit emotional face processing during antidepressant treatment in major depressive disorder. Int. J. Neuropsychopharmacol. 16, 2195–2208. doi: 10.1017/S146114571300062X 23809145

[ref64] Wang X. Öngür D. Auerbach R. P. Yao S. (2016). Cognitive vulnerability to major depression: view from the intrinsic network and cross-network interactions. Harv. Rev. Psychiatry 24, 188–201. doi: 10.1097/HRP.0000000000000081 27148911 PMC4859203

[ref65] Willinger D. Häberling I. Ilioska I. Berger G. Walitza S. Brem S. (2024). Weakened effective connectivity between salience network and default mode network during resting state in adolescent depression. Front. Psych. 15:1386984. doi: 10.3389/fpsyt.2024.1386984, 38638415 PMC11024787

[ref66] Yaden D. B. Haidt J. Hood R. W. Vago D. R. Newberg A. B. (2017). The varieties of self-transcendent experience. Rev. Gen. Psychol. 21, 143–160. doi: 10.1037/GPR0000102

[ref67] Yan G. Zhang Y. Wang S. Yan Y. Liu M. Tian M. . (2024). Global, regional, and national temporal trend in burden of major depressive disorder from 1990 to 2019: an analysis of the global burden of disease study. Psychiatry Res. 337:115958. doi: 10.1016/j.psychres.2024.115958, 38772160

[ref68] Yang X. H. Tian K. Wang D. F. Wang Y. Cheung E. F. C. Xie G. R. . (2017). Anhedonia correlates with abnormal functional connectivity of the superior temporal gyrus and the caudate nucleus in patients with first-episode drug-naive major depressive disorder. J. Affect. Disord. 218, 284–290. doi: 10.1016/j.jad.2017.04.053, 28478357

[ref69] Yen H. Y. Hsu H. Huang W. H. (2024). Virtual reality natural experiences for mental health: comparing the effects between different immersion levels. Virtual Real. 28, 1–11. doi: 10.1007/S10055-024-00958-5

[ref70] Zhang D. He Z. Chen Y. Wei Z. (2016). Deficits of unconscious emotional processing in patients with major depression: an ERP study. J. Affect. Disord. 199, 13–20. doi: 10.1016/j.jad.2016.03.056, 27057648

[ref71] Zhang J. W. Howell R. T. Razavi P. Shaban-Azad H. Chai W. J. Ramis T. . (2021). Awe is associated with creative personality, convergent creativity, and everyday creativity. Psychol. Aesthet. Creat. Arts 18, 209–221. doi: 10.1037/ACA0000442

[ref72] Zhao L. Bo Q. Zhang Z. Li F. Zhou Y. Wang C. (2024). Disrupted default mode network connectivity in bipolar disorder: a resting-state fMRI study. BMC Psychiatry 24:428. doi: 10.1186/S12888-024-05869-Y 38849793 PMC11157927

[ref73] Zhao C. Noordewier M. K. van Elk M. (2026). Effects of awe on self-transcendence: a registered report study. J. Exp. Soc. Psychol. 122:104839. doi: 10.1016/J.JESP.2025.104839

